# Catecholamine-Induced Inflammasome Activation in the Heart Following Photothrombotic Stroke

**DOI:** 10.1007/s12975-024-01311-3

**Published:** 2024-11-18

**Authors:** Xavier O. Scott, Nadine A. Kerr, Juliana Sanchez-Molano, Juan Pablo de Rivero Vaccari, Roey Hadad, Alicia De La Cruz, H. Peter Larsson, W. Dalton Dietrich, Robert W. Keane

**Affiliations:** 1 Department of Molecular Physiology and Cellular Biophysics, University of Miami Miller School of Medicine, Miami, FL USA; 2 The Miami Project to Cure Paralysis, University of Miami Miller School of Medicine, 1095 NW 14th Terrace, Miami, FL 33136 USA; 3 Department of Neurological Surgery, University of Miami Miller School of Medicine, Miami, FL USA; 4 Department of Biomedical and Clinical Sciences, Linköping University, Linköping, Sweden

**Keywords:** Photothrombotic stroke, Catecholamines, Inflammasome, Pyroptosis

## Abstract

Cerebrovascular stroke patients exhibit an increased incidence of cardiac arrhythmias. The pathomechanisms underlying post-traumatic cardiac dysfunction include a surge of catecholamines and an increased systemic inflammatory response, but whether inflammasome activation contributes to cardiac dysfunction remains unexplored. Here, we used a mouse model of photothrombotic stroke (PTS) to investigate the role of inflammasome activation in post-stroke cardiac dysfunction by catecholamines and to evaluate the effectiveness of the inflammasome inhibitor IC100 on inflammasome activation. To evaluate functional electrophysiological changes in the heart by catecholamine treatment, we recorded action potential duration in excised zebrafish hearts with and without IC100 treatment. We show that PTS induced AIM2 inflammasome activation in atria and ventricles that was significantly reduced by administration of IC100. Injection of epinephrine into naïve mice induced a significant increase in AIM2, IL-1b and caspase-8 in atria. Treatment of excised zebrafish hearts with epinephrine shortened the action potential duration and this shortening that was reduced by IC100. These findings indicate that stroke initiates a catecholamine surge that induces inflammasome activation and pyroptosis in the heart that is blocked by IC100, thus providing a framework for the development of therapeutics for stroke-related cardiovascular injury.

## Introduction

Stroke is a leading cause of death and disability; the Centers for Disease Control and Prevention (CDC) report that annually 795,000 people in the USA have a stroke with the economic burden exceeding 50 billion dollars between 2018 and 2019 [1]. Ischemic stroke accounts for most stroke cases in the USA. In ischemic stroke, a reduction in blood flow limits the absorption of glucose and oxygen in the surrounding tissue, leading to spreading depolarizations, increased intracellular calcium, increased extracellular glutamate, neuroinflammation and programmed cell death. Furthermore, increased risk of neurological injury and systemic dysfunction after stroke often results in intensive care admission resulting in high hospitalization costs [2].

Cardiac complications are among the leading causes of mortality and morbidity following a cerebrovascular stroke, second only to acute neurological injury [3]. Nearly 70% of patients with acute ischemic stroke have acute cardiac dysfunction such as arrhythmias, electrocardiogram changes, and myocardial ischemic-like conditions [4–6]. These complications, known as neurocardiogenic syndromes account for over 1.5 million deaths worldwide [7]. The pathological mechanisms regulating cardiac complications following stroke have not been clearly delineated; however, a surge of catecholamines released after stroke and increased systemic inflammation have been suggested to play a role in cardiac injury [8].

Catecholamines are classified as either neurotransmitters or circulating hormones depending on whether the location of synthesis is the sympathetic nervous system or the adrenal medulla, respectively [9]. Norepinephrine is the main catecholamine to function under normal physiological conditions; in contrast, epinephrine is responsible for altered heart rate, force of contraction, and blood flow, as well as the uptake of glucose, oxygen and potassium [10]. Importantly, overactive stimulation of cardiac β-adrenergic receptors by catecholamines has been shown to be toxic to the heart [11, 12].

Inflammasome activation leads to processing of the pro-inflammatory cytokines IL-1β and IL-18 by caspase-1, and plays an important role in the inflammatory response associated with CNS injury, including stroke [13–15] in rodents and humans [16–18]. Furthermore, the inflammasome has also been implicated in cardiovascular disease [18–21], and inflammasome proteins have been shown to affect insulin sensitivity, lipid metabolism, and ion channel activity in cardiomyocytes [22–25]. However, whether catecholamines activate inflammasomes in the heart after stroke has not yet been tested.

Due to the phenotypic similarity between zebrafish cardiac mutants and human diseases, zebrafish have been used as models to dissect the molecular causes of cardiovascular disease in humans [26–30]. Thus, such phenotypic similarities indicate that zebrafish can be used in cardiac research [31]. In a screen of biologically active small molecules for their effects on zebrafish heart rate, it was found that many compounds caused bradycardia [31]. A portion of these identified compounds are known to cause QT prolongation in humans, supporting the feasibility of using zebrafish as a model organism to evaluate drug effects on the cardiovascular system in vivo. In addition, some compounds are known modulators of ion channels or downstream signaling pathways in mammals consistent with the idea that ion flux is critical in regulating cardiac function. These studies offer additional verification that the fish heart responds to pharmacological agents in a similar manner as mammalian models [32].

In this study, we investigated a mechanism by which focal cerebral ischemia results in an inflammatory response mediated by the inflammasome in the brain and provide insights as to a potential mechanism by which catecholamines activate the inflammasome in the heart following cerebral ischemia using a photothrombotic stroke (PTS) model in mice as well as electrophysiological measurement of action potential duration (APD) in ex vivo zebrafish hearts.

## Methods

### Animals and the PTS Model

#### Animal Ethics Declaration

All animal procedures were approved by the Institutional Animal Care and Use Committee (20–158, 20–183) of the University of Miami and in accordance with the ARRIVE guidelines. Animals were housed under standard conditions with a 12-h light/dark cycle and had access to food and water ad libitum.

B6129 male (4 to 6 months old) mice underwent photothrombotic stroke to reproducibility produce focal ischemic damage in the cerebral cortex. This model originally developed in rats induces a well demarcated cortical infarction through a photoactivation of the light sensitive dye (Rose Bengal) previously delivered into the blood stream [33, 34]. The dye-light interactions produce oxygen radicals leading to endothelial damage, subsequent platelet adhesion and aggregation, reducing local cerebral blood flow [35]. More recently, this model has been successfully translated to mice where the light source is applied on the intact skull with no need of craniotomy leading to a reproducible and noninvasive procedure [33, 36–38].

In this study, we used the photothrombotic model so that we could produce consistent patterns of focal ischemic damage produced by local vascular thrombosis in a non-invasive strategy within a predetermined cortical area of the cerebral cortex. Briefly, mice were anesthetized using ketamine (100 mg/kg) and xylazine (10 mg/kg), and an incision in the skin covering the skull was made along the midline to provide access to the intact skull. Rose Bengal (Sigma), a photochemical dye, was injected intravenously through the tail vein at a final concentration of 15 mg/ml, with a dosage of 10 μl/g of animal weight. Next, the skull was irradiated for 9 min 28 mW with a 3-mm beam using a YAG laser (Laserglow), positioned over the right cerebral cortex (−2 mm from Bregma) to induce a closed-head photothrombotic cerebral infarction. Throughout the procedure, the mice were placed on a feedback-controlled heating pad to maintain their body temperature at a normothermic level. The photothrombotic cerebral infarction was induced by positioning the laser over the right cerebral cortex, which includes a large portion of the sensorimotor cortex, approximately −2 mm from Bregma, according to the mouse brain atlas by Franklin and Paxinos [39]. Mice used for histological assessments were allowed to survive for 1 month post-PTS after which the mice were euthanized and the heart was collected for further processing and analyses. All other mice were allowed to survive for 24 h post-PTS and the right (ipsilateral) cortex, heart and plasma were collected. Sham-operated animals underwent all surgical procedures and received rose Bengal injection, but they were not exposed to irradiation. To help alleviate discomfort, a single dose of 0.1 mg/kg subcutaneous slow-release buprenorphine was administered for pain management purposes.

### Epinephrine Treatment and IC100 Administration

B6129 male mice received either 0.5 mg/kg epinephrine (Sigma) or saline through a tail vein injection. In addition, a subset of mice that underwent the PTS received tail vein injections of IC100 [40] at 30 mg/kg (Zyversa Therapeutics, Inc.) or saline control 30 min after PTS. All these animals were sacrificed 24 h after PTS.

### Immunoblot Procedures

Cerebral ipsilateral cortices were collected and snap-frozen using liquid nitrogen. Additionally, the heart was collected after opening the chest cavity and then snap-frozen using liquid nitrogen. Prior to preparing protein lysates, hearts were bisected by excising the heart two-thirds up from the apex to separate the atria from the ventricles. To prepare tissue lysate the cortex, atria, and ventricles were homogenized using protein lysis buffer as described in [41]. For immunoblot analysis of inflammasome signaling proteins, protein lysates were resolved in 4–20% Criterion TGX Stain-Free precast gels (Bio-Rad), using antibodies (1:1000 dilution) against AIM2 (Novus), IL-1β (1:500, Cell Signaling), apoptosis-associated speck-like protein containing a caspase-recruitment domain (ASC) (Santa Cruz), Caspase-1 (Novus Biological), Caspase-8 (Novus Biological) as described in [41]. Polyvinylidene difluoride (PVDF) membranes were imaged using the ChemiDoc Touch Imaging System (BioRad).

### Partial ASC Speck Purification

Atria and ventricle protein lysates were processed for ASC speck isolation as described [42]. Briefly, lysates were filtered using a 5-μm polyvinylidene difluoride membrane for 5 min at 2000 × g. The filtered supernatant was further centrifuged for 8 min at 5000 rpm, the resultant pellet was resuspended in 3-((3-cholamidopropyl) dimethylammonio)−1-propanesulfonate (CHAPS) buffer. Next, subsequent centrifugation at 5000 rpm was performed to pellet ASC specks, which were then resuspended in CHAPS containing disuccinimidyl suberate (DSS). Isolated ASC oligomers were then resolved by immunoblotting using an antibody against ASC (Santa Cruz) as described above.

### IL-1β ECLIA

An Electrochemiluminescent Immunoassay (ECLIA) was run to quantify in pg/mL the concentration of IL-1β using the V-plex kit (MSD) as previously described [43]. Briefly, protein samples were collected from human cardiac aorta-vascular smooth muscle cells in tissue culture and lysates from atria and ventricles were mixed with diluent into a 96-well plate and allowed to incubate overnight. Next, the 96-well plate was washed 3 × with wash buffer followed by addition of detection antibody against IL-1β, and incubated for 2 h prior to washes. Plate was read in the MESO QuickPlex SQ120 (MSD).

### Zebrafish Heart Isolation Procedure

The zebrafish is a model organism that can be used to study heart function and other cardiac processes with the added benefit of investigating electrophysiology questions within the whole organ. These fish are relatively easy to maintain and though zebrafish only maintain a two chambered heart, the cardiac conduction system is strikingly similar to the mammalian cardiac conduction system [44]. All studies with zebrafish were conducted in accordance with the National Institute of Health Guide for the Care and Use of Laboratory animals. 9- to 12-month-old male adult TAB wild-type zebrafish (*n* = 6) were purchased from COX Science Center of the University of Miami and maintained in temperature (26–28 °C) and pH (6.8–7.5) controlled conditions. The collection of the ex vivo hearts used to quantify the ventricular action potential (AP) using optical recordings was completed as described in [45]. Briefly, zebrafish were euthanized using ice-cold water submersion followed by transection of the spinal cord. The zebrafish was then placed in a 60-mm petri dish filled with chilled external solution (in mM: 140 NaCl, 4 KCl, 1.8 CaCl_2_, 1 MgCl2, 10 glucose, and 10 HEPES. pH adjusted to 7.4). Next, the zebrafish was pinned to the SYLGARD184 precoated layer of the petri dish to prepare for heart excision. The pectoral fin was removed along with the surrounding tissue to expose the heart. Once the heart was excised, it was placed in warm external solution with the myosin II inhibitor (-) -blebbistatin (20 μM, Cayman Chemical, RRID:SCR_008945, Ann Arbor MI, USA) to reduce beating artifacts.

### Action Potential Optical Recording and Analysis

Action potential (AP) parameters were analyzed as previously described [45]. Briefly, the zebrafish heart was incubated with Cytovolt1 (also known as Di-4-ANBDQBS, 20 μM; Potentiometric Probes, Connecticut, USA) and FLUO-4FF (2 µM, Life Technologies) for 60 min. Both dyes were diluted in pre-warmed external solution with 20 μM of (-)-blebbistatin at 28 °C. The AP optical recordings were collected using a MacroFluo Leic microscope paired with a Leica APOZ6 zoom and a 5x/0.5 LWD objective. Acquisition of the data was made possible by a photodiode (UDT sensors Inc., Hawthorne, CA, USA) connected to an Axopatch 200B amplifier and an Axon Digidata 1550B digitizer (Molecular Devices, San Jose, CA, USA). Zebrafish excised hearts were paced at 1 Hz (30 V) with a two-electrode system and a Grass S-9 stimulator. Ventricular action potential recordings following exposure to epinephrine at 30 μM, 300 μM and 3 mM were collected every 20 min. The concentration was based on similar studies with the observation that epinephrine likely has less affinity for its receptor in culture and in zebrafish. Recordings were collected from control conditions using only external solution and recording dyes. From each recording, 10 AP were averaged. Action potential duration (APD) were studied at 25, 50, 80, and 90% repolarization as previously described [45].

### Statistical Analyses

Prism 9 software (GraphPad) was used for all statistical analyses. Statistical comparisons between two groups were conducted using a two-tailed Student’s *t*-test after data fulfilled the criteria of normal distribution and equal variance; otherwise, a Mann–Whitney *U*-tests was deployed. One-way ANOVA with Tukey’s multiple comparison tests was carried out for multiple group comparisons. *P*-values of significance used were < 0.05. Outcome measures were evaluated by individuals blinded to experimental groups.

## Results

### Inflammasome Proteins and IL-1β Levels are Increased in the Atria and Ventricle Following PTS

**Fig. 1:**
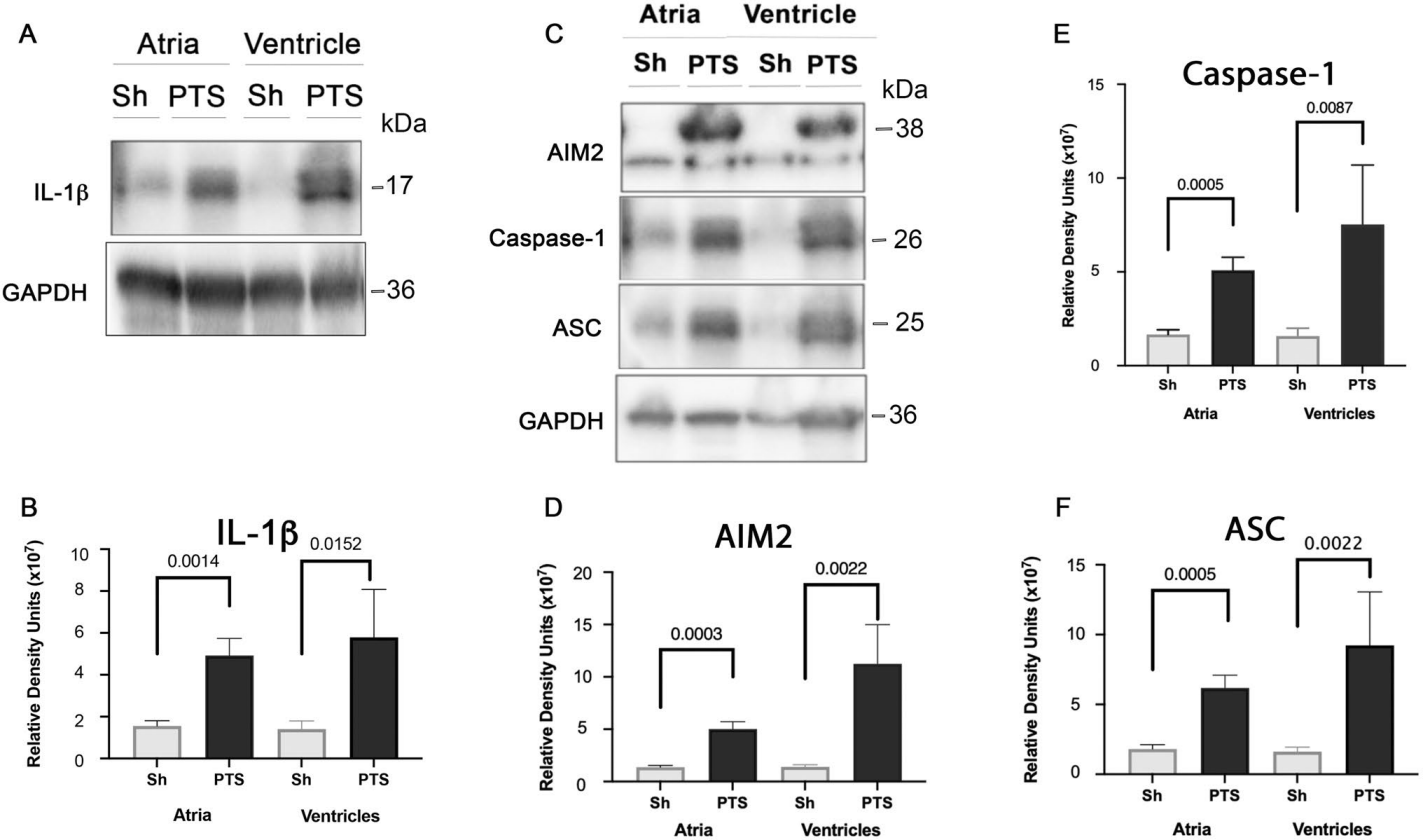
Levels of IL-1β and inflammasome signaling proteins are elevated in the heart following PTS. **A** Representative immunoblots of heart lysates and quantification show significantly increased levels of IL-1β in atria and ventricles of mice 24-h post PTS. **B** 24-h post PTS the expression of IL-1β is statistically elevated in both chambers of the heart (atria and ventricle) compared to sham mice. **C** Representative immunoblots and quantification showing expression of inflammasome signaling proteins in lysates of atria and ventricles from animal at 24 h after PTS for AIM2, Caspase-1, ASC and loading control, GAPDH. **D**–**F** 24-h post PTS the expression of AIM2, Caspase-1, ASC is statistically elevated in atria and ventricles compared to sham mice. Data shown as mean + / − SEM, *p*-values shown for Mann–Whitney *U* statistical test, *N* = 6 per group

To establish whether the pro-inflammatory cytokine IL-1β was elevated in the heart of mice that underwent PTS, we performed immunoblot analysis of atria and ventricle lysates from animals that were sacrificed 24 h after PTS (Fig. 1A). Levels of IL-1β in both atria and ventricles were significantly elevated compared to sham-operated mice (Fig. 1B). Next, atrial, and ventricular lysates were analyzed for levels of inflammasome proteins AIM2, ASC and caspase-1 at 24-h post-PTS (Fig. 1C). Lysates from PTS mice showed significant elevation in the protein levels of AIM2 (Fig. 1D), caspase-1 (Fig. 1E) and ASC (Fig. 1F) in the atria and ventricles compared to sham-operated mice. These data indicate that PTS induces a significant elevation of inflammasome proteins that results in increased IL-1β in the heart acutely after cerebral cortical infarct.

### Mice Subjected to PTS Express Higher Levels of ASC Specks in the Atria and Ventricles

**Fig. 2:**
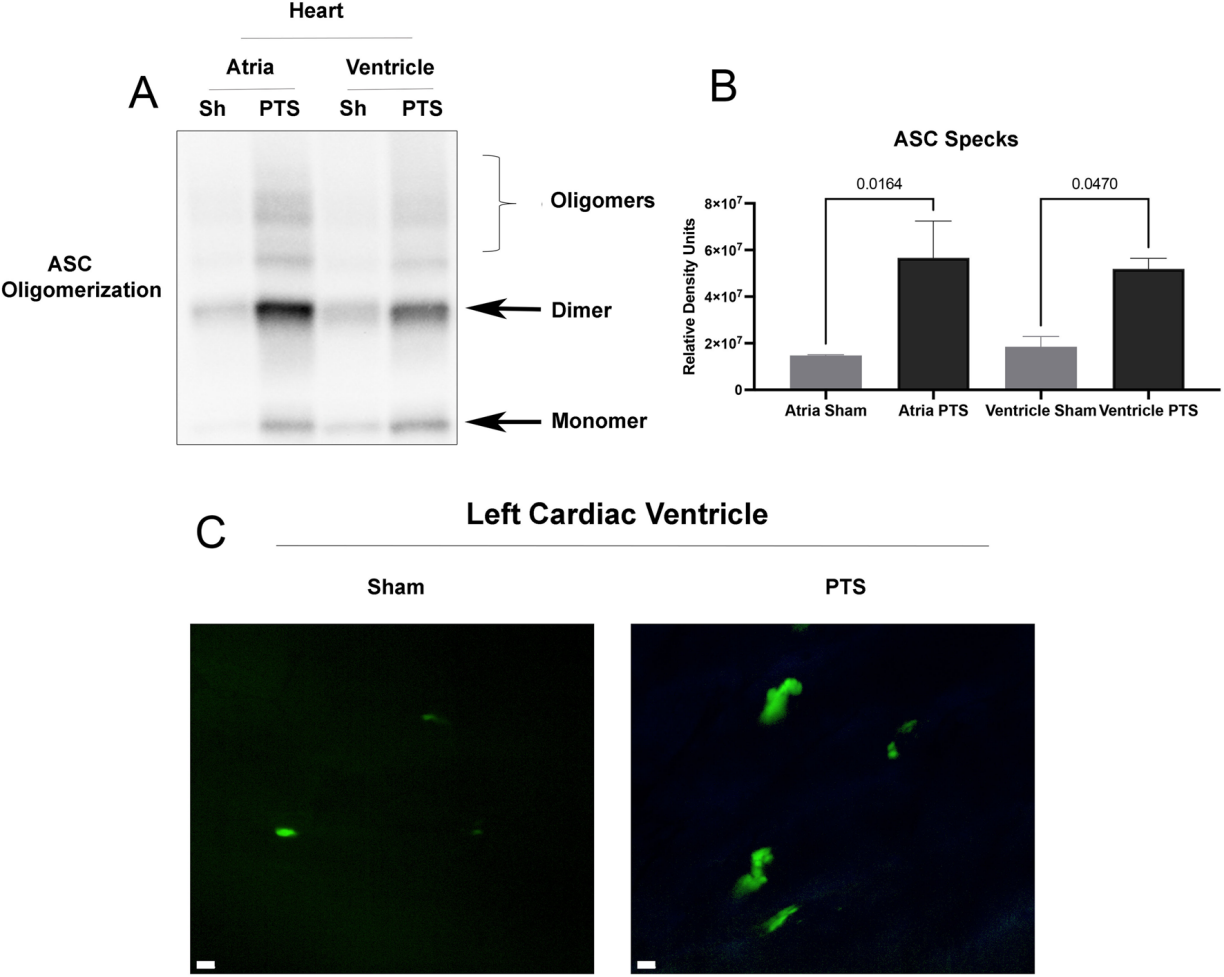
ASC speck levels in cardiac tissue are higher in ASC-citrine reporter mice following PTS. **A** Representative immunoblot of partially-purified ASC specks in cardiac tissue following PTS. **B** Quantification of immunoblots shows significant increase in ASC oligomerization in both heart chambers (atria and ventricle) following PTS. *N* = 3 per group, *p*-values shown are for One-way ANOVA. **C** ASC specks are increased in the ventricles of ASC-citrine reporter mice at 24 h post-PTS**.** *N* = 3 per group. Scale bar = 5 μm

To provide additional evidence for inflammasome activation in the heart following focal cerebral ischemia, we utilized ASC-citrine reporter mice that present fluorescent ASC specks following inflammasome stimulation in vivo [46]. ASC specks were increased in the atria and ventricles (Fig. 2C) of PTS mice compared to sham, indicating increased inflammasome activation in the brain after cerebral ischemia. Furthermore, to establish whether cerebral ischemia induces ASC oligomerization, we isolated ASC specks from atrial and ventricular lysates following PTS (Fig. 2A). ASC laddering was evident and ASC monomers, dimers, and oligomers were observed at ∼25 kDa, ∼50 kDa, and above 75 kDa, respectively (Fig. 2B), indicating ASC speck formation that serves as a platform for caspase-1 activation.

### IC100 Treatment Blocks IL-1β in Atria and Ventricles Following PTS

**Fig. 3:**
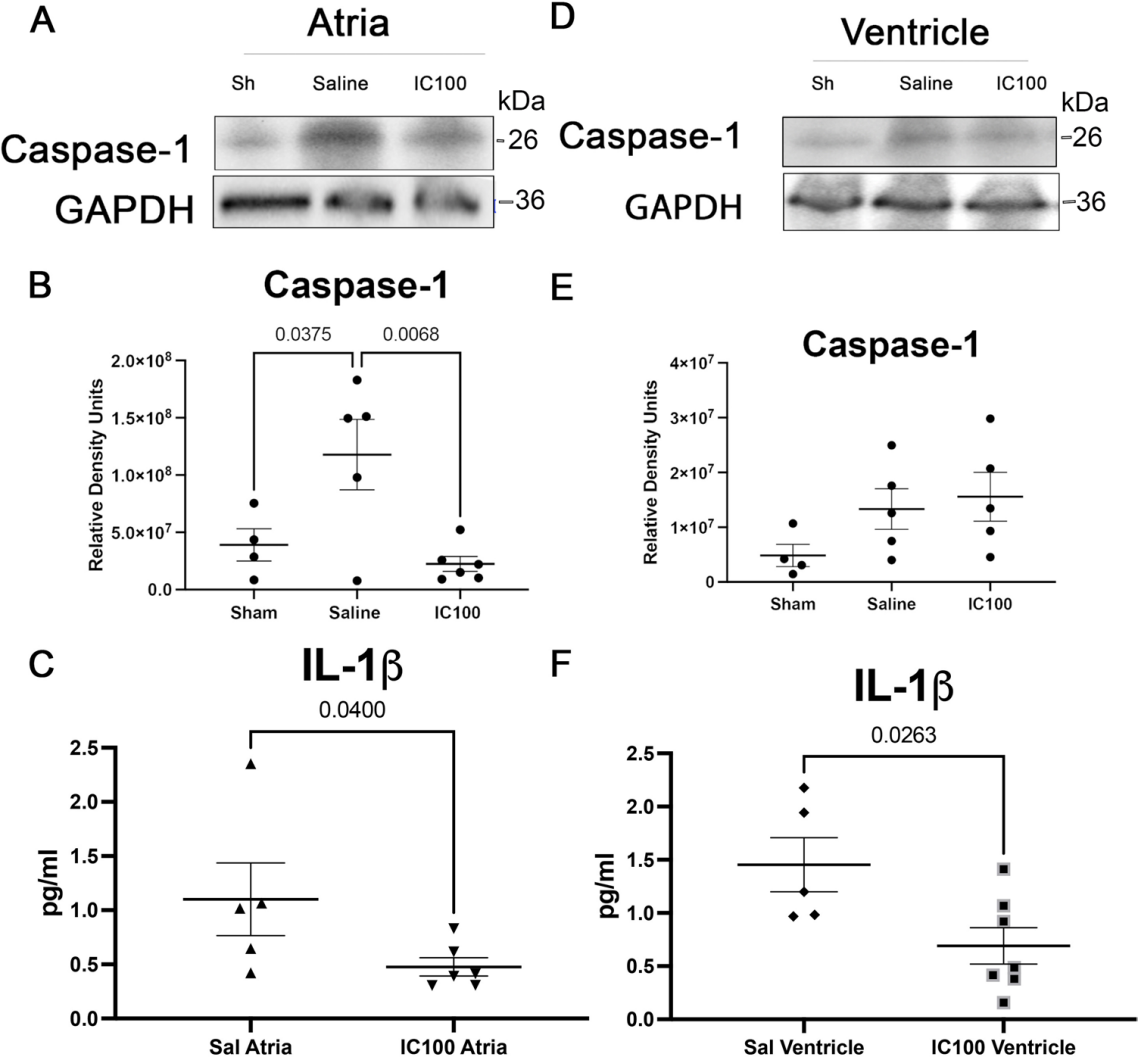
Caspase-1 and IL-1β are reduced in the heart following IC100 administration after PTS. **A** Representative immunoblots of atria lysates and quantification indicate significantly reduced levels of caspase-1 in mice injected with IC100 24 h post-PTS. **B** 24 h post-PTS, caspase-1 is statistically reduced in the atria of IC100-treated mice compared to saline-treated mice. Data shown as mean + / − SEM. *p*-values shown for Student’s *T* test. *N* = 4 to 5 per group. **C** IC100 decreased IL-1β in the atria after PTS. **D** Representative immunoblots of ventricle lysates and quantification showing no difference between saline or IC100 treated mice 24 h post PTS. **E** 24-h post-PTS, the expression of caspase-1 did not differ in the ventricle compared to saline-treated mice. **F** Ventricle lysate expression of IL-1β was reduced in in IC100-treated mice 24 h post-PTS. (B) Data shown as mean + / − SEM. *N* = 4 to 6 per group

Since focal cerebral ischemia induces inflammasome activation in the heart, we next determined whether inflammasome activation in the heart could be blocked by IC100, a humanized monoclonal antibody against ASC. Immunoblot analysis of atria **(**Fig. 3A**)** and ventricles (Fig. 3B) treated with IC100 (30 mg/kg) resulted in a significant reduction in the levels of caspase-1 (Fig. 3B and E) as well as IL-1β (Fig. 3C and F) in the atria of mice compared to sham-operated mice. Additionally, IC100 treatment also significantly reduced the protein levels of IL-1β in the ventricles (Fig. 3F), but the levels of caspase-1 (Fig. 3E) were not significantly altered by the antibody treatment. Together, IC100 was effective in significantly reducing the levels of IL-1β in both chambers of the heart following PTS.

### Epinephrine Treatment Alters Levels of IL-1β and Inflammasome Protein Expression in Atria and Ventricles

**Fig. 4:**
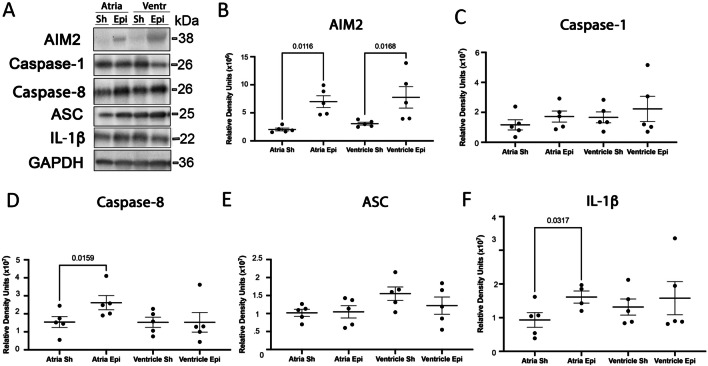
Mice injected with epinephrine exhibit higher levels of AIM2, Caspase-1 and IL-1β in the heart at 24 h after catecholamine treatment. **A** Representative immunoblot and quantification showing expression of (**B**) AIM2, **C** Caspase-1, **D** Caspase-8, **E** ASC and (**F**) IL-1β in atria and ventricles of mice 24 h after epinephrine (0.5 mg/kg) treatment. GAPDH was used as a protein loading control and internal standard. Twenty-four hours after epinephrine injection, the levels of AIM2, Caspasae-8 and IL-1β were increased by catecholamine delivery. Data shown as mean + / − SEM. *p*-values are shown for Mann–Whitney *U* statistical test. *N* = 4 to 5 per group

To establish whether the catecholamine surge contributes to cardiac damage and dysfunction after PTS, we directly injected epinephrine (0.5 mg/kg) into the tail vein of mice and measured levels of inflammasome signaling proteins AIM2, caspase-1, caspase-8, ASC and IL-1β in the heart at 24 h post-injection (Fig. 4A). Although epinephrine treatment did not alter levels of caspase-1 (Fig. 4C) and ASC (Fig. 4E), it did significantly elevated AIM2 in atria and ventricles (Fig. 4B) whereas it significantly increased caspase-8 (Fig. 4D) and IL-1β (Fig. 4F) only in the atria, suggesting a potential role for epinephrine in activating an AIM2 non-canonical inflammasome in the atria and ventricles.

### Epinephrine Exposure Elicits APD Shortening in Zebrafish Heart

**Fig. 5:**
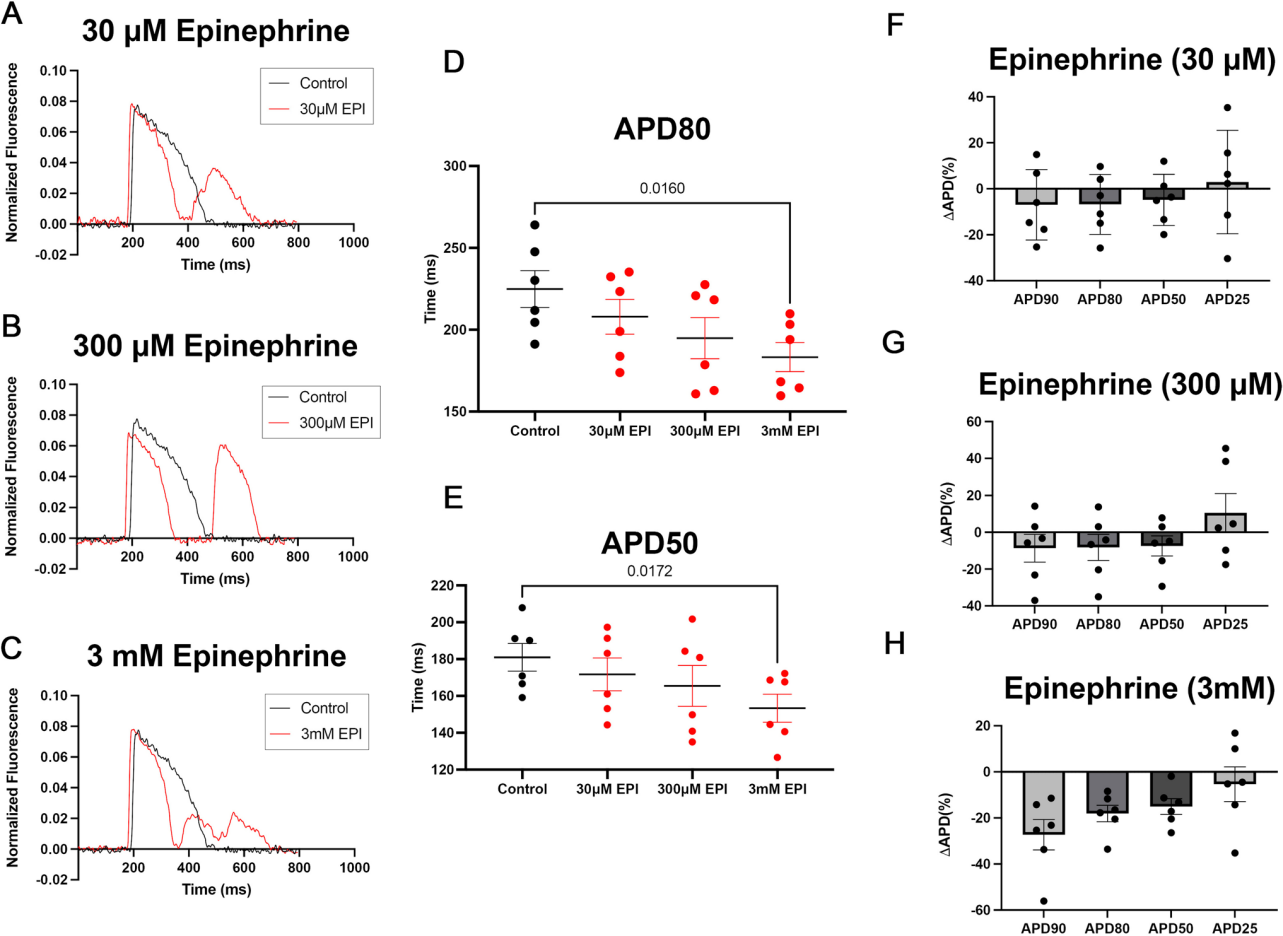
Epinephrine treatment (3 mM) shortens the ventricular corrected action potential duration (APD) in excised zebrafish hearts. **A**–**C** Representative ventricular action potential traces taken from an excised zebrafish heart exposed to various doses of epinephrine (30 μM, 300 μM, 3 mM). **D** 3 mM epinephrine exposure significantly reduces the APD by 80% and APD 50 (**E**) by 50%**.** Data are shown as Mean + / − SEM, *p*-value shown are from One-way ANOVA statistical test, *N* = 6. **F**–**H** Depict the percent change for APD 90, 80, 50, and 25 by concentration of epinephrine (**F**) 30 μM, **G** 300 μM, **H** 3 mM

To study the effect of epinephrine on cardiac action potentials we measured corrected action potential durations (APD) at 25, 50, 80, and 90% repolarizations of excised zebrafish hearts. Corrected APD were used in this study to correct for heart rate. APD25 and APD50 demonstrate the effect of epinephrine on the early phase of the AP, whereas APD80 demonstrates the effect of epinephrine on the entire repolarization process. APD90 reflects the end of repolarization. Treatment with epinephrine at 30 μM, 300 μM, and 3 mM reduced the action potential duration (Fig. 5A–H). However, only epinephrine treatment of 3 mM was statistically significant in reducing the APD at 50 and 80% repolarization (Fig. 5D, E). These results indicate that the shortening effect induced by epinephrine (3 mM) influences the early phase (APD50) and the entire phase (APD80) of the AP, suggesting that exposure to epinephrine at high concentrations has an effect on the ion channel flux that contributes to the ventricular action potential.

### IC100 Attenuates Epinephrine-Induced APD Shortening

**Fig. 6:**
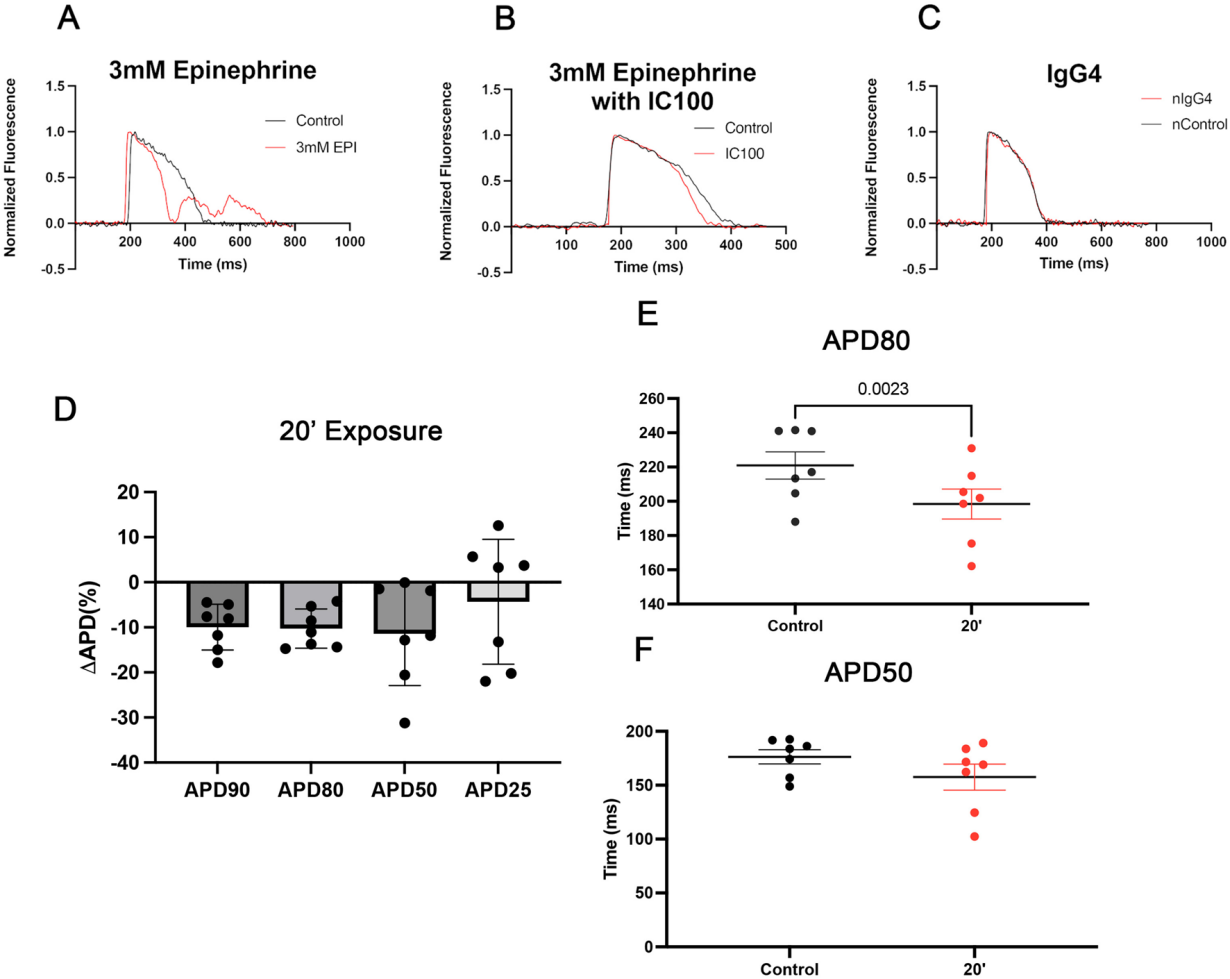
IC100 treatment attenuates epinephrine induced corrected APD shortening. **A**–**C** Representative ventricular action potential traces taken from an excised zebra fish heart exposed to an epinephrine (3 mM), epinephrine with IC100 10 (μg/ml), and epinephrine with IgG4 isotype control (10 μg/ml) at 20’ after treatment. **D** Percent change for corrected APD 90, 80, 50 and 25. **E**, **F** 3 mM Epinephrine reduces the APD 80 and APD 50. Data are shown as Mean + / − SEM, *p*-value shown are from One-way ANOVA statistical test, *N* = 7

**Fig. 7:**
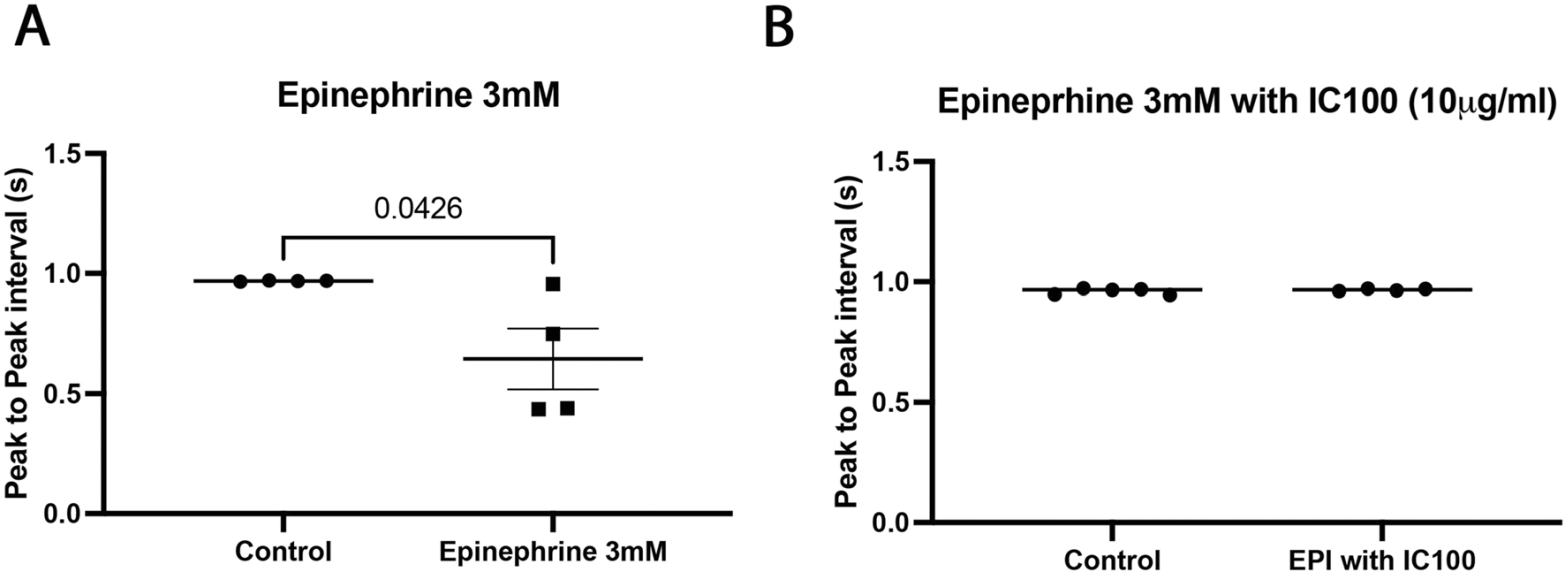
IC100 treatment attenuates the action of epinephrine on the peak-to-peak intervals in excised zebrafish hearts. **A** Quantification of the peak-to-peak interval observed in excised zebrafish hearts at 20 min after exposure to epinephrine. **B** Quantification of the peak-to-peak interval in excised zebrafish hearts after exposure to epinephrine and treatment with IC100. Data are shown as Mean + / − SEM, *p*-value shown are from paired Students *T* test, *N* = 4–5

We have shown that epinephrine both activates the inflammasome complex in cardiac tissue and that it also alters the corrected APD50 and APD80 repolarization. We have also shown that IC100 reduces the activation of the inflammasome complex after PTS. We next tested whether IC100 (10 μg) prevented the APD shortening observed in excised zebrafish hearts exposed to 3 mM epinephrine (Fig. 6B). IC100 reduced the APD shortening by epinephrine, whereas treatment with the IgG4 isotype control has no effect (Fig. 6C). Corrected APD50 was no longer significantly reduced by 3 mM epinephrine when treated with IC100, and the shortening of APD 80 was reduced from 41.6 to 22.5 ms with IC100 treatment (Fig. 6D, E, F). These findings suggest that IC100 interferes with the effect of epinephrine on the AP. Thus, IC100 reduces the epinephrine-induced shortening of APD80 by approximately one-half. In addition to the reduced effect of epinephrine on the APD with IC 100 treatment, the change in the peak-to-peak interval following exposure to epinephrine was attenuated with IC100 treatment (Fig. 7).

## Discussion

Cardiac complications after stroke increase the risk of mortality and morbidity and account for up to one-third of deaths in stroke-survivors [8, 47, 48]. In this study, we show that PTS in mice results in activation of the AIM2 inflammasome in the heart resulting in significant increases in IL-1β and ASC oligomerization, which contributes to the systemic inflammatory response affecting the heart after focal cerebral ischemia in mice. Moreover, administration of epinephrine to naïve mice significantly elevated AIM2 and caspase-8. This finding is consistent with the observed increased protein levels of IL-1β acutely in the heart after PTS. Together, these results that inflammasome activation contributes to the systemic inflammatory response following cerebral infarction in mice. Importantly, treatment with IC100 (30 mg/kg) at 30 min post-PTS significantly reduced the levels of inflammasome proteins and IL-1β in both chambers of the heart. These findings are important in that they show for the first time that PTS initiates catecholamine-induced inflammasome activation that results in cardiac inflammation. In addition, we show that epinephrine-induced shortening of the APD in zebrafish hearts is reduced by IC100.

DAMPs, including various heat-shock proteins, are released by dying brain cells after ischemic stroke [49]. In addition, injured astrocytes, neurons, and oligodendrocytes release brain-derived antigens such as glial fibrillary acidic protein, S100, and myelin basic protein [50]. These DAMPs and brain-derived antigens can pass the ruptured blood–brain barrier (BBB) and enter the systemic circulation [50]. The catecholamine surge after stroke is capable of excessive β-adrenergic stimulation and is known to induce cardiomyocyte necrosis and necroptosis mediated by the RIPK-RIPK3-MLKL pathway [51]. However, it is not clear how catecholamines induce inflammasome activation in the heart.

Several studies have investigated the effects of catecholamines on inflammasome activation. Human monocytes stimulated with phenylephrine show significant elevation in IL-1b production and increased NLRP1 gene expression compared with the unstimulated control samples [52]. Moreover, human monocyte-derived macrophage treated with catecholamines results in priming the NLRP3 inflammasome and induction of production of IL-1β [53]. In contrast, it has been reported that the catecholamine dopamine inhibits canonical and non-canonical inflammasome activation in human microglia [54]. However, there is a scarcity of information about the effects of norepinephrine on inflammasome activation in animal models of stroke. It is possible that catecholamines bind to adrenergic receptors and increase intracellular cyclic adenosine monophosphate (cAMP) that lead to phosphorylation of L-type calcium channels, which could promote mitochondrial calcium overload [8, 55]. Catecholamine exposure increases cytosolic and mitochondrial calcium in cardiomyocytes, which disrupts the mitochondrial membrane potential. Mitochondrial overload leads to an increase in reactive oxygen species (ROS) and release of mtDNA which have been shown to activate the NLRP3 and AIM2 inflammasomes [56–59]. The combination of mitochondrial dysregulation and alterations in ion balance may activate the inflammasome in the heart, resulting in myocardial damage. However, further work is needed to decipher the precise molecular pathway for inflammasome activation by catecholamines in the heart.

Inflammasome activation has been documented in a myriad of cardiovascular diseases. In stroke, inflammasome signaling proteins are found to be elevated in clots, extracellular vesicles, and serum of stroke patients compared to controls [16, 18]. Mice that received PTS showed evidence of both inflammasome activation and pyroptosis not only in the cerebral cortex but also in the gut [60]. Furthermore, middle cerebral artery occlusion (MCAO), another experimental model of stroke used in rodents has been reported to produce elevated levels of AIM2 inflammasome protein complex and downstream signaling proteins, caspase-1, ASC, and Gasdermin-D, providing evidence of pyroptosis in the CNS through the AIM2 inflammasome after cerebrovascular occlusion [61]. On the other hand, recent evidence has accumulated that suggests the involvement of inflammasome complexes in disrupted or failing heart and systemic complications after stroke. For example, adenosine triphosphate (ATP) and dsDNA, which activate inflammasomes, are released from cardiomyocytes in ischemic events [62–65]. A landmark study, the Canakinumab Anti-inflammatory Thrombosis Outcome Study (CANTOS) trial highlighted the importance of IL-1β as a key therapeutic target for cardiovascular diseases [66–68]. Interestingly, AIM2 and NLRC4 were found elevated in cardiac lymphoid cells of failing human hearts; while expression of AIM2 was found in an experimental rodent model of heart failure, further suggesting a role of the inflammasome in the context of adverse cardiac remodeling and heart failure [66].

Immune system activation, leading to inflammation after stroke is an important factor in the pathophysiology of stroke progression. The complex interplay of the local and systemic inflammatory responses after stroke involves many immune cell types and circulating signals and is a significant contributor to potential infections and other secondary complications after ischemia [69, 70]. Following cerebral ischemia, there is increased infiltration of leukocytes into cardiac tissue as well as activation of the complement pathway [71–73]. There is also evidence of cross-talk between inflammatory responses and sympathetic activation in ischemic and hemorrhagic stroke [74], and the pro-inflammatory cytokines released by damaged neuronal and glial cells stimulate the posterior hypothalamus to increase sympathetic output and circulating catecholamine levels [8]. Interestingly, the catecholaminergic stress that takes place after brain ischemia coincides with influx of inflammatory cells into the heart that is associated with cardiac damage after stroke [8, 75]. However, in the PTS model, we did not observe a significant infiltration of immune cells into the heart following injury, indicating that the inflammatory response is primarily associated with inflammasome activation in cardiomyocytes.

Electrically excitable cells may exhibit changes in membrane potential that may lead to problems in excitation–contraction coupling and altered cardiac rhythm. Here, we show that catecholamines induced shortening of the APD duration in the zebrafish heart. In human hearts, catecholamines increase heart rate by augmenting the cAMP-responsive hyperpolarization-activated cyclic nucleotide-gated channel 4 pacemaker current (I_f_) and by promoting inward Na^+^/Ca^2+^exchanger current (I_NCX_) by a “Ca^2+^ clock” mechanism in sinoatrial nodal cells (SANCs) [76]. Moreover, β-AR agonist stimulation increases heart rate in part by enhancing SAN cAMP and PKA activity and increasing *I*_f_. Future studies are needed to decipher the precise physiological mechanisms by which catecholamines affect heart rate in the zebrafish model.

Our work demonstrates that epinephrine treatment produces differential effects on the levels of IL-1β and inflammasome protein expression in atria and ventricles. We observed that epinephrine treatment did not alter the levels of caspase-1 and ASC, but significantly elevated AIM2 in atria or ventricles and significantly increased the levels of IL-1β and caspase-8 in the atria. Furthermore, increases in catecholamines in the blood may have differential effects on the two chambers of the heart due to regional innervation differences that influence inflammasome activation. Alternatively, it is possible that differences in the vascular supply between the two chambers may influence levels of inflammasome activation. Thus, further studies are needed to explain the regional variation of inflammasome protein increases in the heart induced by catecholamines.

 Lastly, we used a therapeutic monoclonal antibody (mAb) (IC100) against the pyrin domain of ASC that has been shown to diminish pathological inflammasome activation in spinal cord injury [77], traumatic brain injury [78, 79], acute lung injury [80], multiple sclerosis [81], and inflammaging [40, 81]. Treatment of zebrafish excised hearts with IC100, prior to epinephrine exposure, resulted in a reduced shortening of the action potential duration by epinephrine compared to the saline-treated group. Sequence analysis has shown that zebrafish ASC (zASC) have a high sequence homology to mammal orthologs, with 35% sequence identity and 51% similarity to human ASC (hASC) [82]. Thus, the monomeric structure and function of ASC are highly conserved in zebrafish. Further studies are needed to determine whether IC100 interferes with zASC assembly and IL-1β cleavage and to determine the precise mechanism by which IC100 interferes with inflammasome signaling and the effects on zebrafish heart rate. However, this highly specific biologic has a wide tissue distribution, including the heart [40] and acts both intracellularly and intercellularly making it an attractive therapeutic option for several disease modalities, including inflammation and cardiovascular diseases [40, 83]. Therefore, IC100 or other treatment strategies that successfully target inflammasome signaling may prove to be a therapeutic option for the treatment of a variety of inflammatory conditions affecting the heart. Taken together, this study reveals catecholamine-induced cardiac inflammasome activation following cerebrovascular stroke; thus, providing a novel mechanism by which inflammasomes contribute to systemic inflammation after cerebral ischemia.

## Data Availability

The data supporting the findings of this study are available from the corresponding author upon request.
